# Design of UAV-Embedded Microphone Array System for Sound Source Localization in Outdoor Environments †

**DOI:** 10.3390/s17112535

**Published:** 2017-11-03

**Authors:** Kotaro Hoshiba, Kai Washizaki, Mizuho Wakabayashi, Takahiro Ishiki, Makoto Kumon, Yoshiaki Bando, Daniel Gabriel, Kazuhiro Nakadai, Hiroshi G. Okuno

**Affiliations:** 1 Department of Systems and Control Engineering, School of Engineering, Tokyo Institute of Technology, 2-12-1 Ookayama, Meguro-ku, Tokyo 152-8552, Japan; gabriel@ra.sc.e.titech.ac.jp (D.G.); nakadai@ra.sc.e.titech.ac.jp (K.N.); 2 Graduate School of Science and Technology, Kumamoto University, 2-39-1 Kurokami, Chuo-ku, Kumamoto 860-8555, Japan; k.washizaki@ick.mech.kumamoto-u.ac.jp (K.W.); m.wakabayashi@ick.mech.kumamoto-u.ac.jp (M.W.); takahiro.ishiki@lixil.com (T.I.); kumon@gpo.kumamoto-u.ac.jp (M.K.); 3 Graduate School of Informatics, Kyoto University, Yoshida-honmachi, Sakyo-ku, Kyoto 606-8501, Japan; yoshiaki@kuis.kyoto-u.ac.jp; 4 Honda Research Institute Japan Co., Ltd., 8-1 Honcho, Wako, Saitama 351-0188, Japan; 5 Graduate Program for Embodiment Informatics, Waseda University, 3-4-1 Okubo, Shinjuku-ku, Tokyo 169-8555, Japan; okuno@nue.org

**Keywords:** robot audition, sound source localization, multiple signal classification, outdoor-environment measurement, real-time measurement, unmanned aerial vehicle

## Abstract

In search and rescue activities, unmanned aerial vehicles (UAV) should exploit sound information to compensate for poor visual information. This paper describes the design and implementation of a UAV-embedded microphone array system for sound source localization in outdoor environments. Four critical development problems included water-resistance of the microphone array, efficiency in assembling, reliability of wireless communication, and sufficiency of visualization tools for operators. To solve these problems, we developed a spherical microphone array system (SMAS) consisting of a microphone array, a stable wireless network communication system, and intuitive visualization tools. The performance of SMAS was evaluated with simulated data and a demonstration in the field. Results confirmed that the SMAS provides highly accurate localization, water resistance, prompt assembly, stable wireless communication, and intuitive information for observers and operators.

## 1. Introduction

Research on remote sensing techniques involving unmanned aerial vehicles (UAV) is important to improve search and rescue in disaster-stricken areas because such technologies enable prompt action regardless of the terrain. Search and rescue tasks with UAV rely mainly on vision, which is vulnerable to poor lighting conditions or occlusions. A UAV-embedded microphone array system is expected to be effective for the detection of people needing assistance in disaster-stricken areas. Since a UAV-embedded microphone array system receives rotor and wind noise as well as environmental sounds, the target sound is contaminated by ego-noise and other noise. Sound source processing should be able to localize, and discriminate a target sound from noise. Robot audition software [1,2,3] has been developed to cope with a mixture of sounds contaminated by noise. In particular, the open source robot audition software HARK (Honda Research Institute Japan Audition for Robots with Kyoto University) [4,5] provides noise-robust sound processing functions: sound source localization, source separation and recognition of separated sounds. Basic technologies of robot audition have been developed for use in indoor environments, and it is necessary to advance these technologies for use in outdoor environments, such as for search and rescue tasks using UAV. Five main challenges to developing such a system for UAV include:

1. sound source localization;
2. sound source separation and sound enhancement;
3. sound source classification;
4. real-time processing and intuitive visualization tools;
5. robustness of the device in outdoor environments.

The first challenge has been addressed in recent years in several studies including as a main research topic to find people in disaster situations, e.g., localization of an emergency signal from a safety whistle [6], and that of speech with a low signal-to-noise ratio (SNR) [7,8,9]. To locate the source of a sound, algorithms based on multiple signal classification (MUSIC) [10] are often used because they can effectively localize sound sources in highly noisy environments. In particular, MUSIC based on incremental generalized singular value decomposition with correlation matrix scaling (iGSVD-MUSIC-CMS) developed by Ohata et al. demonstrated good performance under dynamically changing noise [9]. iGSVD-MUSIC-CMS could localize sound sources in a low SNR environment, −15 dB. There is a severe trade-off between the speed and performance of the signal processing. Since only offline processing was reported in their evaluation, evaluation in real time is necessary for application to search and rescue tasks.

The second challenge, in order to identify a target sound source in extremely noisy environments, is also important, in two goals: to improve SNR of the target sound and to improve intelligibility of the separated signals. The first goal of the present study was to improve sound source classification (the third challenge). Recent studies on restoration of distorted signals have been reported including: sound source separation with a linear process [11] and integrated frameworks of sound source separation and classification using end-to-end training [12,13]. While the first goal targeted machine listening, the second goal targets human listening, that is, an operator tries to identify a target sound source manually, e.g., by inspecting sound spectrograms or by listening to separated sounds. In this case, intelligibility is a primary requirement. This second goal has not been reported as a function for UAV, although it is important to the UAV operator.

The third challenge, to effectively discriminate a target sound source, such as a human-induced sound, from other sound sources has been investigated [11,12,13], albeit these studies only reported offline processing and did not mention real-time processing.

Regarding the fourth challenge, latency in visualizing flight and sound source information should be as short as possible for efficient operation of UAV. Since UAV operators may be situated far from the UAV, visualization tools should be capable of displaying accurate information regarding UAV location and sound source. Finally, regarding the fifth challenge, to ensure system efficiency, all-weather acoustic sensors and reliability of wireless communication using a Wi-Fi signal that carries acoustic signals, which are necessary in outdoor environments, should be proven.

In this paper, we report the development of a UAV-embedded microphone array system that resolved four of the above five challenges. The third challenge, sound classification, which we regard to be at a higher level than the other four, will be investigated in a separate study. The remaining of the paper is organized as follows: Section 2 describes the design method and details of the UAV-embedded microphone array system. Section 3 evaluates and discusses the performance of the system. Section 4 is the conclusion.

## 2. Methods

### 2.1. Design of Water-Resistant Microphone Array for Use Onboard UAV

To address the first and fifth challenges in Section 1, we designed and developed a microphone array.

**Figure 1:**
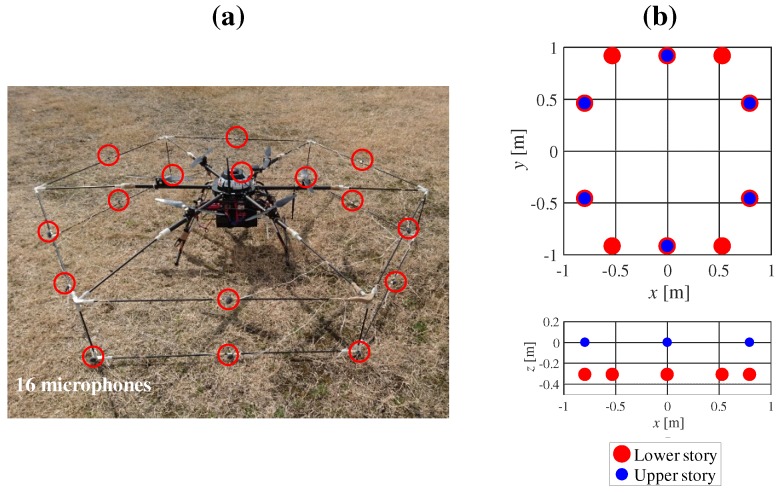
HMAS (hexagonal microphone array system). (**a**) the 16 microphones marked as red circles; (**b**) coordinates of the microphone positions in the HMAS.

**Figure 2:**
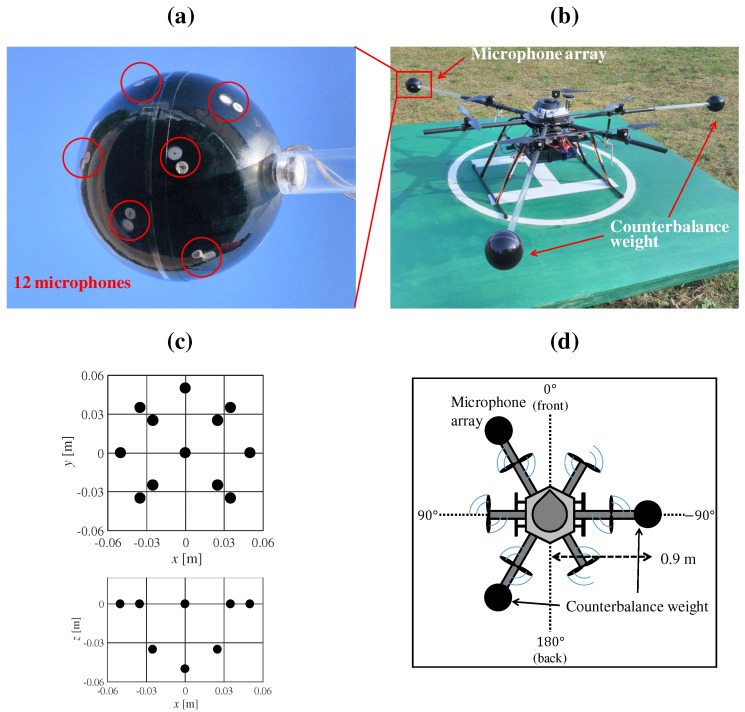
SMAS (spherical microphone array system). (**a**) the 12 microphones, and six of them marked as red circles; (**b**) UAV (unmanned aerial vehicles) with SMAS and two counterbalance weights; (**c**) coordinates of the microphone positions in the SMAS; (**d**) layout of the SMAS and two counterbalance weights in the UAV.

**Figure 3:**
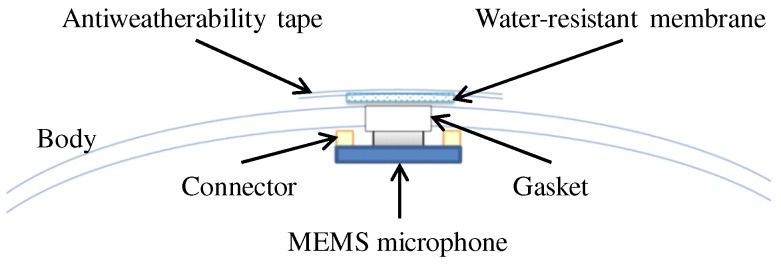
Internal structure of the SMAS.

Figure 1 shows our prototype hexagonal microphone array system (HMAS). Sixteen redMEMS (Micro Electro Mechanical Systems) microphones are set on a two-story hexagonal frame whose diagonal length is 1.8 m. The microphones and cables, being exposed, were vulnerable to water, and risk of disconnection. Additionally, the complexity of the frame demanded a lot of time for assembly of the HMAS. To solve these problems, we designed a spherical microphone array system (SMAS), which is water resistant and simple to assemble in a UAV (Figure 2). As shown in Figure 2a or Figure 2c, twelve MEMS microphones are embedded in a spherical body with a diameter of 0.1 m. Since a single strut and one cable connects the array to the UAV, its assembly is simple and the risk of disconnection is reduced. Unlike with the HMAS, where the microphones are equidistant around the UAV, the weight of the UAV is unbalanced with the SMAS. To solve this, we added two weights, each of the same size and mass, to counterbalance the SMAS. As shown in Figure 2d, the SMAS and weights are set at intervals of $120^{\circ }$, and the direction of the SMAS is $30^{\circ }$ on the UAV coordinates. Figure 3 shows internal structure of the SMAS. To embed microphones into the body, gaskets were used. The microphone was attached to the gasket so that holes of the microphone and the gasket were coincident. When there is a gap between the microphone and the body, the microphone cannot receive acoustic signals precisely because of reverberations in the body. To fill gaps between microphones and the body, ring-shaped connectors were used. To ensure water resistance of the SMAS, holes of gaskets were covered with a water-resistant membrane and an antiweatherability tape. Since a water-resistant membrane and an antiweatherability tape are enough thin to pass acoustic signals through, they do not influence signals received by microphones.

### 2.2. Stabilization of Wireless Communication

To resolve the fifth challenge, to stabilize wireless communication, we incorporated a high-gain antenna that could receive a Wi-Fi signal from the UAV, which carries acoustic signals at a ground station. In addition, a communication protocol was also implemented to improve robustness.

For sound source localization, acoustic signals recorded by SMAS on the UAV are sent via wireless communication using a Wi-Fi signal to a ground station, and processed by a computer. A network system was constructed by assuming that the distance between the UAV and the ground station is short. However, in an outdoor environment, the network communication has the potential to be unstable as the distance increases. To ensure reliable wireless communication, two improvements were made.

**Figure 4:**
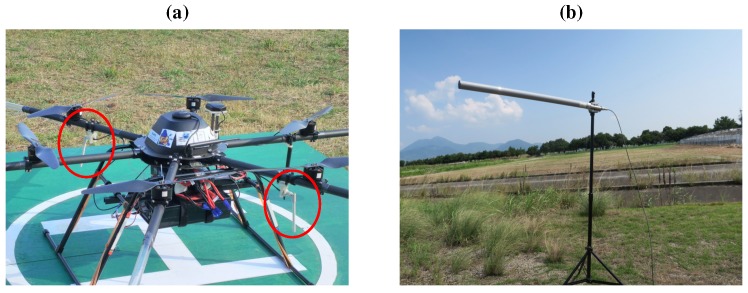
(**a**) antennas on the UAV marked as red circles; (**b**) the Yagi antenna at a ground station.

First, we replaced an antenna at the ground station with the Yagi antenna (FX-ANT-A5, CONTEC (Osaka, Japan)) [14] to improve throughput of communication [15]. For acoustic signals recorded by 12 microphones, throughput of approximately 5 Mbps is necessary. Therefore, a high gain antenna was used for reliable wireless communication in outdoor environments. Figure 4 shows antennas (FX-ANT-A7, CONTEC) [16] on the UAV and the Yagi antenna at a ground station. On the UAV, two antennas was assembled to arms of UAV. At a ground station, the Yagi antenna was set on a tripod.

Second, we changed the communication protocol from TCP (Transmission Control Protocol) to UDP (User Datagram Protocol). In wireless communication tests at tens of meters of distance between the hovering UAV and the ground station, packet loss occurred approximately 400 times per minute. With TCP, each packet loss caused a retransmission request, greatly reducing acoustic signal throughput. Hence, the protocol was changed to UDP. Because UDP provides no guarantee of data integrity, no retransmission request is sent and throughput is maintained. When a packet loss occurs, the ground station receives defective acoustic signals. However, because the minimum frame size for sound source localization is much larger than one packet, the impact is negligible.

### 2.3. Development of Intuitive Visualization Tools for Operators

For the second and fourth challenges, we developed three visualization tools that display information regarding the sound source on the UAV coordinates and acoustic signals before and after their enhancement.

**Figure 5:**
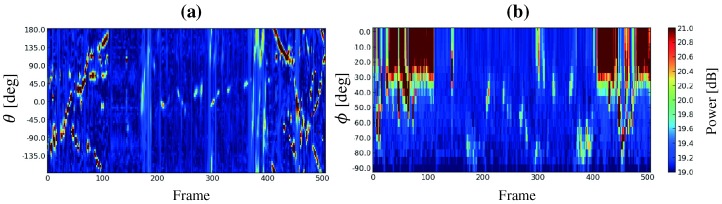
MUSIC (multiple signal classification) spectrum. (**a**) azimuth direction; (**b**) elevation direction.

**Figure 6:**
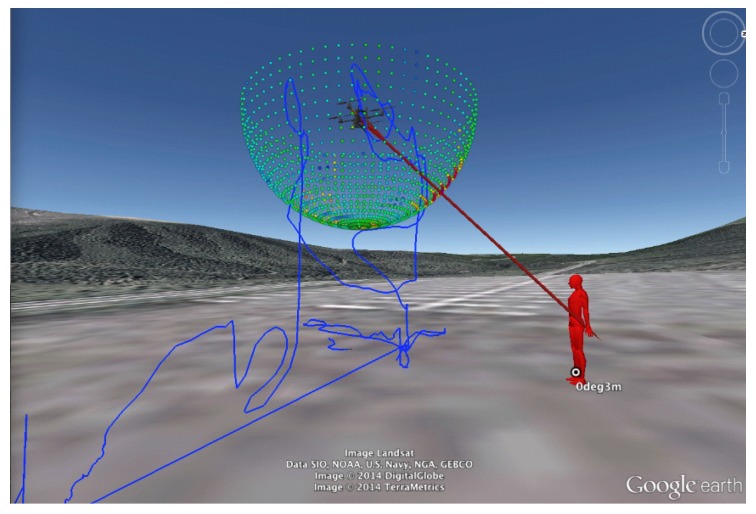
Previous visualization tool based on Google Earth^TM^.

Essential to the system is a visualization tool to display sound source localization results. Several groups have developed such a tool for sound source localization [17,18,19]. Figure 5 shows MUSIC spectra produced by the MUSIC method [10]. It visualizes sound power arriving from each direction. The horizontal axis represents the frame number (time) and the vertical axis represents the azimuth angle θ (**a**) or the elevation angle ϕ (**b**). The sound power is represented as a color map. It is difficult to quickly determine the direction and time of a sound source with the MUSIC spectrum. In order to visualize sound source localization and the UAV location and orientation, we developed a tool to display such data on Google Earth^TM^ (Google (Los Angeles, CA, USA)) (Figure 6) [20]. Because users can change their viewpoint freely on Google Earth^TM^, they can intuitively grasp the situation of the environment, the UAV, and the sound source. However, this tool is for observers only and not for the operator. Unlike an indoor environment, in an outdoor environment, the distance between the UAV and the operator may be large, necessitating a tool for its effective operation. Therefore, we developed visualization tools for operators.

**Figure 7:**
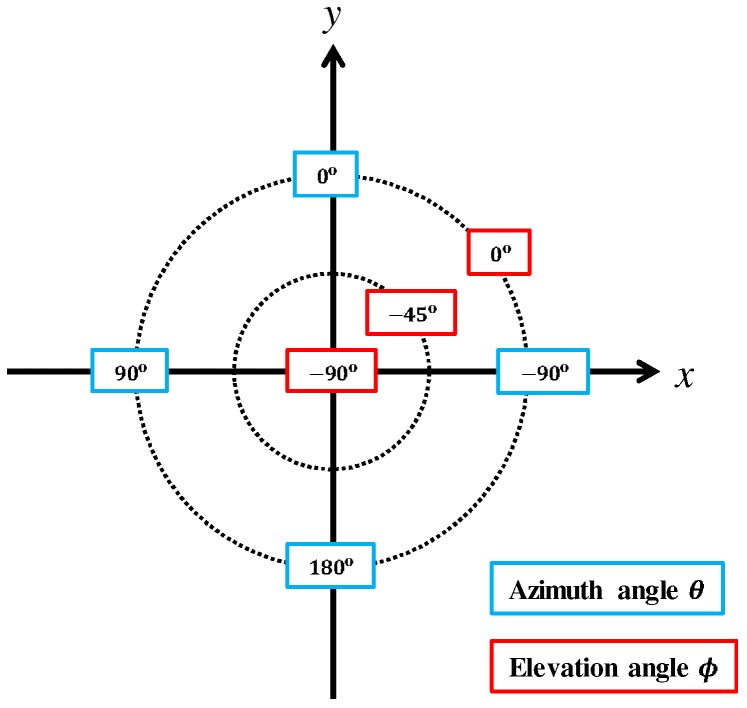
Visualization tool coordinate system.

**Figure 8:**
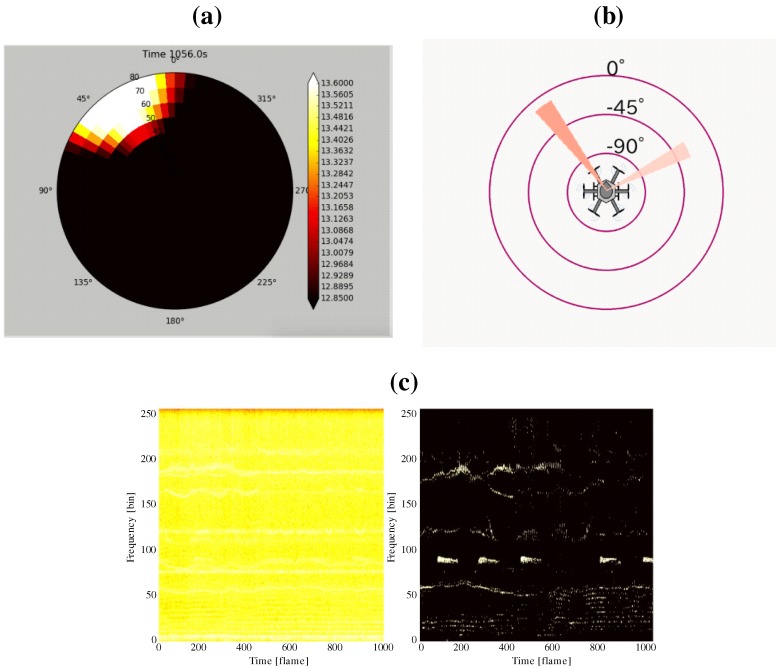
Visualization tools. (**a**) MUSIC spectrum; (**b**) sound direction; (**c**) spectrograms of captured sound (**left**) and after enhancement (**right**).

Because an operator controls the UAV with reference to its coordinates, a user friendly method would be to display sound source directions on the UAV’s coordinates. Therefore, the coordinate system shown in Figure 7 was defined. Forward direction of the UAV is defined as the positive direction of the *y*-axis, and the azimuth and elevation are projected to the circumferential and radial directions, respectively. Using this coordinate system, two visualization tools to display sound source directions were developed as shown in Figure 8. Figure 8a shows the MUSIC spectrum. The sound power in each direction is depicted by a color map. Figure 8b illustrates only the sound directions after threshold processing for the MUSIC spectrum. In addition, spectrograms of the recorded sound and after sound enhancement by online robust principal component analysis (ORPCA) [21] are displayed as in Figure 8c. The left panel shows a spectrogram of the recorded acoustic signal, and the right panel shows it after enhancement. The horizontal and vertical axes represent time and frequency, respectively. By viewing these three sets of data in real time, even when located far from the UAV, the operator knows the relationship between the UAV and the sound source.

### 2.4. Sound Source Localization Method

We used two methods of sound source localization, namely SEVD-MUSIC (MUSIC based on Standard Eigen Value Decomposition), which is an original broadband MUSIC method [10], and iGSVD-MUSIC [9]. SEVD-MUSIC has low noise robustness and low computational cost, while iGSVD-MUSIC has high noise robustness and high computational cost. Either of these can be selected according to the circumstances. Algorithms of SEVD-MUSIC and iGSVD-MUSIC are described below.

#### 2.4.1. SEVD-MUSIC

*M* channel input sound signals of the *f*-th frame are Fourier transformed to Z(ω,f), from which a correlation matrix R(ω,f) is defined as follows:

(1) $$R\left(\omega ,f\right)=\frac{1}{T_{R}}\sum_{\tau =f}^{f+T_{R}-1}Z\left(\omega ,\tau \right)Z^{*}\left(\omega ,\tau \right).$$

ω is the frequency bin index, $T_{R}$ is the number of frames used for the correlation matrix calculation, and $Z^{*}$ is a complex conjugate transpose of Z. The SEVD-MUSIC method calculates eigenvectors through an SEVD of the obtained R(ω,f):

(2) $$R\left(\omega ,f\right)=E\left(\omega ,f\right)\Lambda \left(\omega ,f\right)E^{*}\left(\omega ,f\right).$$

Λ(ω,f) is a matrix with diagonal components that are eigenvalues in a descending order. E(ω,f) is a matrix containing eigenvectors corresponding to Λ(ω,f). Using E, and a transfer function, G(ω,ψ), corresponding to the sound source direction, ψ=(θ,ϕ) in the UAV coordinates, the MUSIC spatial spectrum, P(ω,ψ,f), is calculated:

(3) $$P\left(\omega ,\psi ,f\right)=\frac{|G^{*}\left(\omega ,\psi \right)G\left(\omega ,\psi \right)|}{\sum_{m=L+1}^{M}\left|G^{*}\left(\omega ,\psi \right)e_{m}\left(\omega ,\psi \right)\right|}.$$

*L* is the number of target sound sources, and $e_{m}$ is the *m*-th eigenvector contained in E. P(ω,ψ,f) is average over ω direction to estimate the direction of the sound source:

(4) $$\bar{P}\left(\psi ,f\right)=\frac{1}{\omega_{H}-\omega_{L}+1}\sum_{\omega =\omega_{L}}^{\omega_{H}}P\left(\omega ,\psi ,f\right).$$

$\omega_{H}$ and $\omega_{L}$ are indices corresponding to the upper and lower limits of the used frequency bin, respectively. Threshold processing and peak detection is performed for $\bar{P}\left(\psi ,f\right)$ and ψ of the obtained peak is detected as the sound source direction.

#### 2.4.2. iGSVD-MUSIC

In iGSVD-MUSIC, for the *f*-th frame, the section of the length of $T_{N}$ frames from the $f-f_{s}$-th frame is assumed to be a noise section, and the noise correlation matrix K(ω,f) is calculated:

(5) $$K\left(\omega ,f\right)=\frac{1}{T_{N}}\sum_{\tau =f-f_{s}-T_{N}}^{f+f_{s}}Z\left(\omega ,\tau \right)Z^{*}\left(\omega ,\tau \right).$$

The iGSVD-MUSIC method estimates noise in each frame and responds to dynamic change in noise. The noise component can be whitened by multiplying $K^{-1}$ to R from the left. The iGSVD-MUSIC method calculates singular vectors through the GSVD of $K^{-1}\left(\omega ,f\right)R\left(\omega ,f\right)$:

(6) $$K^{-1}\left(\omega ,f\right)R\left(\omega ,f\right)=Y_{l}\left(\omega ,f\right)\Sigma \left(\omega ,f\right)Y_{r}^{*}\left(\omega ,f\right).$$

Σ(ω,f) is a matrix with diagonal components of singular values in a descending order. $Y_{l}\left(\omega ,f\right)$ and $Y_{r}\left(\omega ,f\right)$ are matrices containing singular vectors corresponding to Σ(ω,f). Then, the MUSIC space spectrum is calculated:

(7) $$P\left(\omega ,\psi ,f\right)=\frac{|G^{*}\left(\omega ,\psi \right)G\left(\omega ,\psi \right)|}{\sum_{m=L+1}^{M}\left|G^{*}\left(\omega ,\psi \right)y_{m}\left(\omega ,\psi \right)\right|}.$$

$y_{m}$ is the *m*-th singular vector contained in $Y_{l}$. P(ω,ψ,f) is averaged over ω direction to estimate the direction of the sound source:

(8) $$\bar{P}\left(\psi ,f\right)=\frac{1}{\omega_{H}-\omega_{L}+1}\sum_{\omega =\omega_{L}}^{\omega_{H}}P\left(\omega ,\psi ,f\right).$$

Threshold processing and peak detection is performed for $\bar{P}\left(\psi ,f\right)$ and ψ of the obtained peak is detected as the sound source direction.

Both sound source localization methods based on MUSIC basically assume an acoustic far-field. However, by using the transfer function *G* according to the distance to sound sources, it is possible to localize sound sources at any distance. In addition, at the altitude at which a UAV flies normally (at least a few meters), an acoustic field is a far-field. Therefore, the accuracy of sound source localization depends on a SNR of an acoustic signal rather than a distance between a microphone array to a sound source.

### 2.5. Structure of Microphone Array System

**Figure 9:**
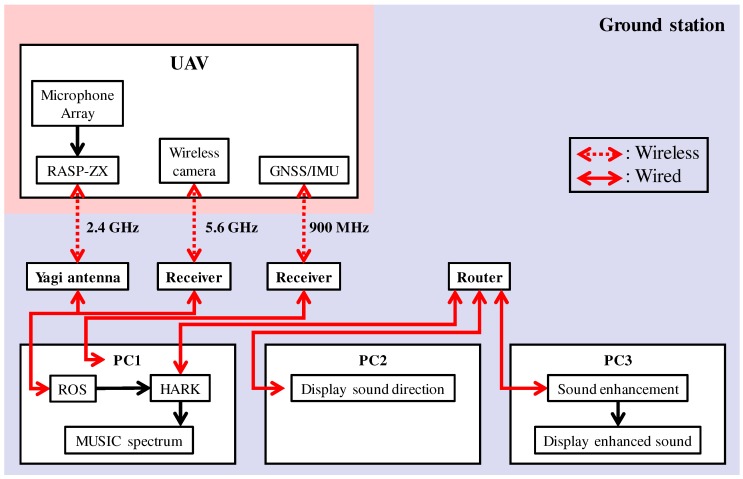
Configuration of SMAS.

By integrating the above components, the microphone array system was constructed. Figure 9 shows the SMAS configuration. The microphone array on the UAV was connected to a multi-channel sound signal recorder, RASP-ZX (System In Frontier (Tokyo, Japan)) [22] for synchronous recording of 12 ch sound signals. The sound signals were recorded at a sampling frequency of 16 kHz, and a quantization bit rate of 24 bits. Recorded acoustic signals, images from the wireless camera and data from a GNSS/IMU (Global Navigation Satellite System/Inertial Measurement Unit) sensor were transmitted through a wireless network to the ground station. Different frequencies were used for the wireless communications to prevent cross talk. In the SMAS, data from a GNSS/IMU sensor and images from the wireless camera were not used; therefore, only recorded acoustic signals were received by the Yagi antenna. The received data was integrated using ROS (Robot Operating System) to provide general versatility. The acoustic signals were processed by a PC using a sound source localization method. HARK was used for the algorithm implementation. The data after processing was shared by three PCs via a router. To reduce the processing load of one computer for real-time visualization, visualization tools were displayed using three laptops. PC1, PC2 and PC3 displayed the MUSIC spectrum (Figure 8a), sound direction (Figure 8b) and enhanced sound (Figure 8c), respectively. Since the SMAS is a separate system from the UAV, including its power supply, it can be applied to various UAVs.

## 3. Results and Discussion

The performance of the SMAS was evaluated using numerical sound simulation and by demonstration in an outdoor environment.

### 3.1. Evaluation Procedure

**Figure 10:**
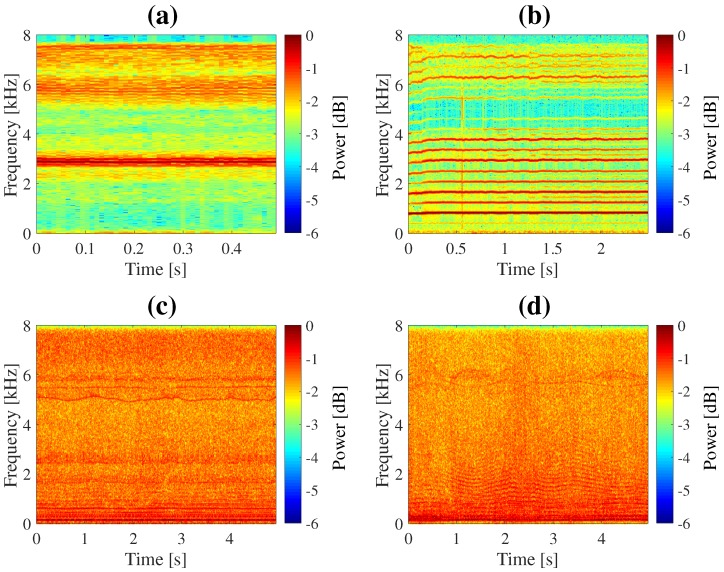
Spectrograms. (**a**) whistle; (**b**) voice; (**c**) noise of UAV recorded by hexagonal microphone array; (**d**) noise of UAV recorded by spherical microphone array.

Sound localization performance was evaluated using acoustic signals created in a numerical simulation. Using transfer functions corresponding to two types (hexagonal and spherical) of microphone array and sound samples, acoustic signals arriving from every direction were created. Recorded noise of an actual flying UAV was added to the created signals. The direction was set as every $5^{\circ }$ in the azimuth range from $-180^{\circ }$ to $180^{\circ }$ and the elevation range from $-90^{\circ }$ to $0^{\circ }$. As sound sources, a whistle and human voice were used. A Mini Surveyor MS-06LA (Autonomous Control Systems Laboratory (Chiba, Japan)) was used as the UAV. Spectrograms of the sound sources and the noise of the UAV recorded by each of the two microphone arrays are shown in Figure 10. Simulated signals were created in different SNR, −20, −10, 0, 10 and 20 dB. Simulated signals were processed by the SMAS and results were evaluated. Performance was also evaluated by demonstration in the field.

### 3.2. Results of Simulation

**Figure 11:**
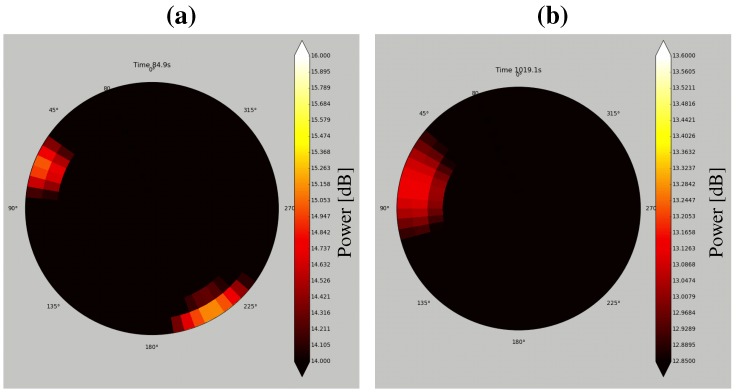
MUSIC spectra. (**a**) SEVD-MUSIC (MUSIC based on Standard Eigen Value Decomposition); (**b**) iGSVD-MUSIC (MUSIC based on incremental generalized singular value decomposition).

**Figure 12:**
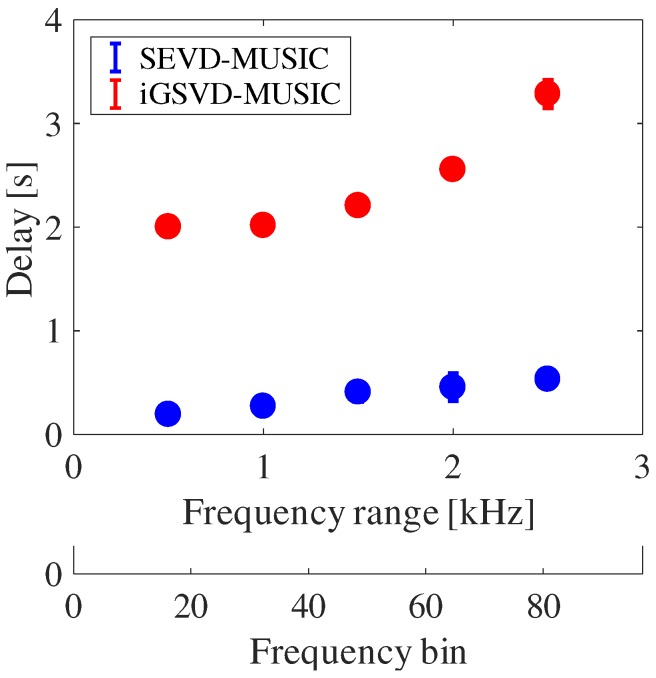
Time delay of the system.

The main differences between SEVD-MUSIC and iGSVD-MUSIC are noise robustness and computational cost. Since its computational cost is low, SEVD-MUSIC has a short delay; however, it has poor noise tolerance. Since iGSVD-MUSIC includes noise whitening, it has noise tolerance but a long delay. Thus, real-time property and noise tolerance are in a trade-off relationship. Figure 11 shows MUSIC spectra processed by SEVD-MUSIC (**a**) and by iGSVD-MUSIC (**b**) using spherical microphone array. The target sound is located around $\theta =80^{\circ }$. In both MUSIC spectra, the target sound source power can be seen. However, in Figure 11a, the noise power of the UAV can also be seen. Figure 12 shows the delay in the system when the frequency range, which is used in the MUSIC method, is changed. The horizontal axis represents the frequency range and the number of the frequency bin ($\omega_{H}-\omega_{L}+1$), and the vertical axis represents delay. As shown in Figure 12, iGSVD-MUSIC has a time delay of 2 to 3 s longer than that of SEVD-MUSIC. In addition, as the frequency range increases, the time delay increases.

**Figure 13:**
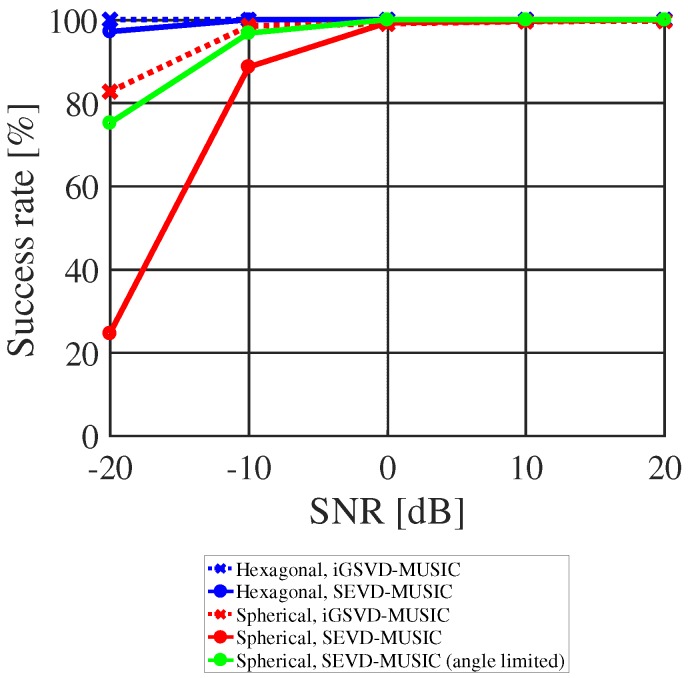
Success rate of localization.

Based on these results, localization performance was evaluated by its success rate. The success rate was calculated based on the UAV coordinates. When the angle of the maximum value of the MUSIC spectrum is matched with a set angle, it is defined that sound source localization succeeded. All simulated sounds were processed using sound source localization method, and the success rate was calculated. Figure 13 shows the success rate of localization for the hexagonal and a spherical microphone arrays, processed by SEVD-MUSIC and iGSVD-MUSIC. The frequency range used in the MUSIC method was from 500 to 3000 Hz. In the hexagonal array, the success rate of both MUSIC methods was almost 100% even with SNR less than 0 dB. In the spherical array, the success rate was lower than that of hexagonal. In particular, the success rate of SEVD-MUSIC was less than 30% when the SNR was −20 dB. This lower success rate was considered due to the smaller aperture diameter in the spherical array at 0.1 m compared to 1.8 m in the hexagonal. Therefore, the detection area of the MUSIC spectrum was limited to increase the accuracy of localization with SEVD-MUSIC. As shown in Figure 11a, noise of the UAV appear in one direction constantly as directional noise, in this case at the azimuth angle of around $-150^{\circ }$. To avoid the effect of such noise, the detection azimuth angle was limited to $-60^{\circ }\leq \theta \leq 120^{\circ }$. The success rate in a case when the detection angle was limited is plotted as the green line in Figure 13. By limiting the detection angle, the success rate of short-delay SEVD-MUSIC using the spherical microphone array with SNR −10 dB could be increased to approximately 97%. Since the SNR, when blowing a whistle or speaking to an actual flying UAV from a distance of around 10 m was approximately −10 to 0 dB, it was considered sufficient localization performance. This technique can be used with microphones located at one site on the UAV, unlike the HMAS in which microphones are dispersed around the UAV. Due to the location of our microphone array, parameters for sound source localization could be easily tuned to attain accurate localization with a small latency.

### 3.3. Results of the Demonstration

**Figure 14:**
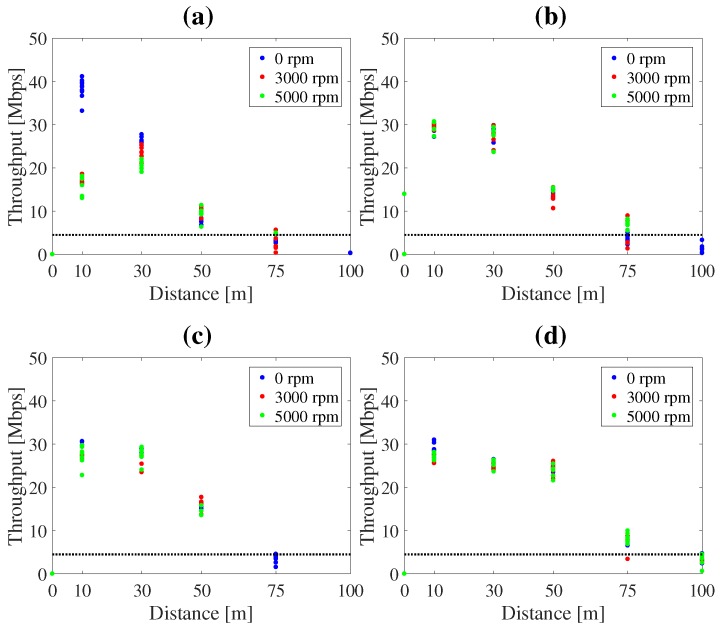
Throughputs by antennas type. (**a**) diversity; (**b**) Yagi (small); (**c**) collinear; (**d**) Yagi (large).

**Figure 15:**
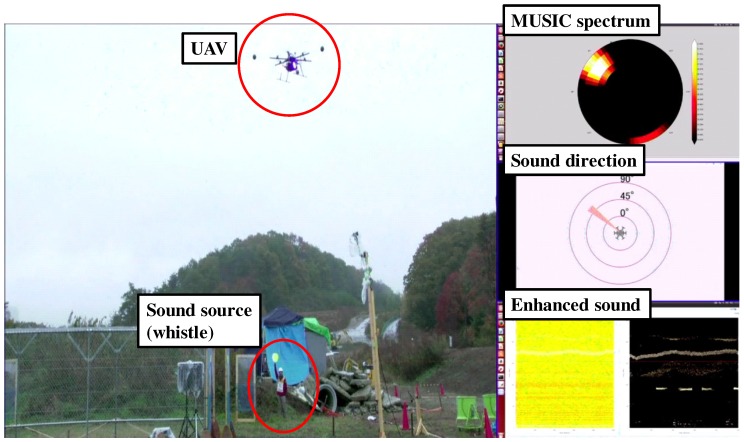
Data visualized in the demonstration.

Regarding efficiency in assembling the system, the HMAS took two hours to assemble, and especially time consuming was assembly of the frame and electric cables. In contrast, the SMAS took 40 min to assemble and 2 min to take off after switching on the UAV. Regarding water resistance, although the demonstration was performed in light rain, the SMAS worked without failure. To assess the reliability of wireless communication, throughputs were compared among four different antennas. Figure 14 shows the results of throughputs by antenna type: Diversity (FX-ANT-A1, CONTEC) [23], small Yagi (FX-ANT-A3, CONTEC) [24], Collinear (FX-ANT-A2, CONTEC) [25] and large Yagi (used in SMAS). Throughputs were measured by fixing the UAV on the ground at distances, 10, 30, 50, 75 and 100 m, with propeller rotation speeds, 0, 3000 and 5000 rpm. The required throughput (5 Mbps) is shown as a dotted line in Figure 14. It was found that throughput surpassed 5 Mbps even at 75 m by using the large Yagi-antenna. In the demonstration, the wireless network worked without disconnecting in the distance of tens of meters. To examine the intuitiveness of visualization tools, camera image, MUSIC spectrum, sound direction, and enhanced sound data were displayed as in Figure 15. These visualization tools provided directions of sound sources and other data in real time for the audience and operator, intuitively.

### 3.4. Discussion

Before the demonstration, we conducted over 10 test flights, and all sound source localization trials were successfully completed. Thus, usability of the SMAS was verified. Table 1 and Table 2 show a summary of pros and cons of each microphone array system and sound source localization method. For the microphone array system, the HMAS provides high accurate localization; however, it does not have water resistance and efficiency in assembling. The SMAS provides lower accurate localization than the HMAS; however, we can increase the accuracy of localization depending on sound source localization method. For sound source localization method, SEVD-MUSIC has low noise tolerance and a small latency, while iGSVD-MUSIC has high noise tolerance and a large latency. Angle-limited SEVD-MUSIC can have high noise tolerance only when microphones located at one site on the UAV like the SMAS. Thus, because of their characteristics, we can select them according to the situation. In the demonstration, sound sources could be localized in real time with high accuracy using the SMAS and angle-limited SEVD-MUSIC because the SNR of the recorded acoustic signal was over −10 dB. However, in order to develop the system for the detection of people in a disaster-stricken area, a new sound source localization method with higher noise robustness and lower computational cost is needed. In addition, since there are several sound sources at an actual site, it is necessary to separate and identify human-related sound from recorded sounds. In future work, we will integrate the proposed sound source identification method using deep-learning [11,12,13] to the SMAS.

## 4. Conclusions

In this paper, we developed a UAV-embedded microphone array system for an outdoor environment. First, a novel microphone array was designed to ensure water resistance and efficiency of assembly. A 12 ch microphone array, including a spherical body of simple structure, was designed. By using coated microphones and a simple structure, water resistance and efficiency of assembly were ensured. Second, the antenna and communication protocol were changed to obtain reliable wireless communication. To improve throughput, the antenna at the ground station was changed to the Yagi antenna. To avoid reducing throughput, the communication protocol was changed from TCP to UDP. Third, intuitive visualization tools for a UAV operator were developed. By integrating the above improvements, the microphone array system was constructed. Tests showed that our microphone array system for an outdoor environment that is independent from the UAV provides highly accurate sound source localization performance in real time, and has effective intuitive operator visualization tools.

## Figures and Tables

**Table 1:** Pros and cons of the HMAS and the SMAS.

	Accuracy of Localization	Water Resistance	Efficiency in Assembling
HMAS	◯	×	×
SMAS	△	◯	◯

**Table 2:** Pros and cons of SEVD-MUSIC, iGSVD-MUSIC and angle-limited SEVD-MUSIC.

	Noise Tolerance	Latency
SEVD-MUSIC	×	◯
iGSVD-MUSIC	◯	×
Angle-limited SEVD-MUSIC	△	◯
